# The Association of Seasonal Malaria Chemoprevention with Educational Outcomes in Senegal, 2013–2019: A District-Level Ecological Analysis

**DOI:** 10.4269/ajtmh.26-0036

**Published:** 2026-10-06

**Authors:** Mohammed Ndiaye, Donal Bisanzio, Amber Gove, Lauren M. Cohee, Richard Reithinger

**Affiliations:** ^1^ University of Maryland, College Park, Maryland, USA;; ^2^ RTI International, Washington, District of Columbia, USA;; ^3^ Department of Veterinary Sciences, University of Turin, Turin, Italy;; ^4^ Tangerine Central, Washington, District of Columbia, USA;; ^5^ University of Maryland School of Medicine, Baltimore, Maryland, USA;; ^6^ Liverpool School of Tropical Medicine, Liverpool, United Kingdom;; ^7^ Pure Earth, New York, New York, USA

## Abstract

Malaria negatively impacts childhood development and education outcomes. However, evidence on the effects of malaria control on education outcomes is limited. Since 2013, Senegal has scaled up routine monthly administration of malaria chemoprevention to young children during the peak malaria transmission season (i.e., seasonal malaria chemoprevention [SMC]) as a critical malaria control intervention. We conducted a district-level ecological analysis to determine whether large-scale implementation of SMC is associated with improved education outcomes. Using generalized additive mixed models applied to routine malaria intervention and education indicator data for the 2013–2019 period, we show that in comparison with non-SMC districts, districts that had received multiple SMC rounds had improved education outcomes, specifically enrollment, dropout and repetition rates, as well as pass rates of children completing primary school. Our findings highlight the broader benefits of SMC beyond malaria prevention and underscore its role in advancing educational attainment and human development goals in malaria-endemic settings.

## INTRODUCTION

Malaria remains a major cause of morbidity and mortality affecting children in sub-Saharan Africa.^1^^,^^2^ Although children under 5 years of age are the population group most at risk of severe malaria and malaria-related mortality, school-age children (SAC) 5 to 15 years old suffer an underappreciated burden of malaria.^2^ It is estimated that in Africa alone 200 million school-age children are at risk of malaria, with prevalence of infection often exceeding 50% in this age group.^3^ Seasonal malaria chemoprevention (SMC) is a critical malaria control intervention,^4^ currently protecting ∼50 million children each year.^5^ Recommended by the WHO since 2012 for countries with seasonal malaria transmission, SMC is the monthly administration of a single dose of sulfadoxine-pyrimethamine and three daily doses of amodiaquine (SP-AQ) during the peak malaria transmission season; depending on the length of the rainy season, 3 to 5 cycles of SMC are conducted.^6^ When effectively deployed, SMC can substantially reduce the incidence of uncomplicated and severe malaria, as well as death.^4^^,^^7^ As of 2025, 20 countries had adopted SMC (i.e., Benin, Burkina Faso, Cameroon, Chad, Côte d’Ivoire, Gambia, Ghana, Guinea, Guinea Bissau, Kenya, Mali, Mauritania, Madagascar, Mozambique, Niger, Nigeria, Senegal, South Sudan, Togo, and Uganda)^1^ at different scales of implementation.

Several studies have shown that malaria can lead to adverse childhood development outcomes, specifically cognition and learning, and affect short-term education outcomes, including attendance, repetition and dropout, and long-term educational attainment (defined as the highest level of education an individual has completed).^2^^,^^3^ The primary objective of our study was to determine whether large-scale implementation of SMC is associated with improved education outcomes. Specific objectives were to assess whether 1) education outcomes were higher in districts where SMC had been administered to children over multiple years, and 2) routine malaria intervention and education data could be used to measure the impact of a malaria intervention such as SMC on childhood development and education attainment outcomes. Thus, we conducted a district-level ecological analysis correlating scale-up of SMC with key education indicators using routine programmatic data.

## MATERIALS AND METHODS

### Intervention.

Senegal introduced SMC in 2013, piloting the intervention in select districts. By 2024, SMC had been scaled up to 16 health districts, primarily in the more malaria-endemic southeastern regions of the country.^8^ In Senegal, SMC targets children 3 to 120 months of age, with SMC implemented in 3 to 5 monthly community-based campaigns (cycles) conducted during the peak malaria transmission season (generally from June/July to October). Since its launch in 2013, the number of SMC cycles has been adapted to better align with Senegal’s transmission patterns. Thus, in 2024, five cycles were conducted in Kédougou, Salémata, Saraya, and Dianke Makhan; four were conducted in Kolda, Médina Yoro Foulah, and Dahra, as well as select health districts in Tambacounda region (Kidira and Goudiry); three cycles were also conducted in the remaining health districts of Tambacounda region (Tambacounda, Koumpentoum, and Makacolibantang).^8^

Seasonal malaria chemoprevention is administered as per national protocol, with the first dose of SP-AQ administered under direct observation by community drug distributors, who distribute SP-AQ door-to-door in each district; the second and third doses are administered by the child’s caregiver. Seasonal malaria chemoprevention administration is accompanied by monitoring efforts, including small-scale household surveys to validate coverage and adherence. Each campaign round is followed by evaluation meetings to inform subsequent implementation.

### Educational system in Senegal.

Senegal’s formal education system consists of preschool (ages 3–5 years), six grades of primary school (ages 6–11 years), as well as secondary school, split between four years of lower (ages 12–15 years) and three years of upper secondary (ages 16–18 years) school. Only primary school is officially compulsory; school enrollment and age of entry vary by region. In 2022, official estimates of primary net enrollment rates (i.e., the percent of primary school-age children enrolled) for grades 1 to 6 was 74.5%, reflecting late entry to school, repetition, and a substantial number of out-of-school children.^9^

### SMC dataset.

Information on SMC implementation was derived from the U.S. President’s Malaria Initiative Malaria Operational Plans and National Malaria Control Program records. The SMC dataset included, by district: 1) the number of SMC cycles, 2) SMC programmatic intervention coverage, and 3) the age range of the children targeted for SMC. We defined a given district’s SMC “exposure” as the cumulative number of years in which at least one round of SMC was conducted between 2013 and 2019. This exposure variable was used as the primary predictor in subsequent statistical analyses examining associations between malaria prevention efforts and education outcomes.

### Education dataset.

We compiled district-level education indicators from 2013 to 2019 annual reports published by the Senegalese Ministry of Education and the Agence Nationale de la Statistique et de la Démographie (ANSD [National Agency of Statistics and Demography]). Datasets included: 1) preschool enrollment rates (noncompulsory, for children ages 3–6 years), 2) primary school dropout and repetition rates (primary education is compulsory and includes grades 1–6), and 3) pass rates on the Certificat de Fin d’Études Élémentaires (CFEE) exam (i.e., a high-stakes national exam that marks the end of primary education and serves as a gateway to secondary education). These indicators were selected for their relevance to education access, progression, and performance. All data were harmonized across years and administrative units to ensure temporal and geographic comparability. Where possible, district-level data were validated using multiple sources, including annual summaries compiled by the Ministry of Education and ANSD.

### Data analysis.

To assess the association between SMC implementation and educational indicators, a generalized additive mixed model (GAMM) was applied.^10^ Generalized additive mixed models are a nonparametric regression technique that allows modeling of nonparametric relationships between a response variable and continuous covariates while accounting for random effects affecting that relationship.^10^ Our GAMM framework used education indicators as dependent variables, year as a nonlinear smoothing term, and SMC exposure (coded by the number of years a district had implemented SMC between 2013 and 2019) as the key independent variable. Region and district were modeled as random effects to control for spatial heterogeneity (including malaria transmission, non-SMC malaria intervention coverage, and education intervention coverage). All analyses were conducted using R software.^11^

## RESULTS

### SMC implementation.

Between 2013 and 2019, SMC coverage expanded across regions in Senegal, with eligible districts receiving SMC for between 1 and 6 years. In total, 16 districts were targeted for SMC during the study period, with increasing number of cycles (monthly administrations) per year of SMC over time in malaria high-burden areas, including districts in Kédougou, Kolda, and Tambacounda regions. Depending on the study year, programmatic district-level SMC coverage was high, ranging from 92.5% to 99.5% (Table 1).

**Table 1:** Summary of SMC intervention and education indicator data in study districts

Variables of Interest	2013		2014		2015		2016		2017		2018*		2019^†^	
	No SMC	SMC	No SMC	SMC	No SMC	SMC	No SMC	SMC	No SMC	SMC	No SMC	SMC	No SMC	SMC
SMC														
Number of regions targeted for SMC	10	4	10	4	10	4	10	4	10	4	–	–	10	4
Number of SMC cycles	NA	2	NA	3	NA	3–4	NA	3–4	NA	3–4	NA	–	NA	3–4
District-level programmatic SMC coverage (mean)	NA	92%	NA	99%	NA	99.5%	NA	97%	NA	94%	NA	–	NA	95%
Age range of children targeted for SMC (years)	NA	<10	NA	<10	NA	<10	NA	<10	NA	<10	NA	–	NA	<10
Education outcome indicators (range across districts)														
Pre-school enrollment (for children 3–6 years of age)	2,700– 56,024	2,397– 9,508	3,031– 56,733	3,056– 9,922	3,466– 60,534	2,908– 10,046	3,568– 64,950	2,811– 12,945	3,506– 70,064	3,159– 14,383	3,909– 72,115	3,081– 14,272	4,216– 75,398	3,323– 14,823
Primary school dropout rate	6.1–19.5%	10.0–14.6%	2.6–15.8%	4.3–14.6%	4.6–15.8%	12.0–15.1%	5.0–14.1%	12.9–15.3%	4.7–17.6%	10.1–16.2%	6.0–20.0%	12.0–14.5%	–	–
Primary school repetition rate	1.9–3.3%	1.8–3.5%	2.2–3.8%	2.1–3.6%	2.1–3.6%	1.1–3.4%	2.8–4.8%	2.2–4.7%	2.9–4.6%	2.3–4.9%	2.8–4.4%	2.2–4.6%	–	–
Pass rate of end-of-primary school graduation exam	19.6–43.3%	19.4–51.2%	20.5–48.7%	14.0–37.0%	20.6–54.4%	24.2–38.2%	51.5–72.1%	38.8–56.9%	49.4–75.7%	48.8–53.2%	43.1–77.9%	48.2–59.0%	–	–

NA = Not applicable; SMC = seasonal malaria chemoprevention.

* A nationwide strike in the health care sector interrupted SMC campaign activities.^8^

^†^ Education rate data were not collected in 2019. SMC was implemented in the following regions: Kedougou, Kolda, Sedhiou, and Tambacounda.

### Education outcomes.

Between 2013 and 2019, descriptive statistics revealed a trend toward improved education indicators over time: student enrollment in preschools (ages 3–6 years) increased steadily, and CFEE success rates improved (Table 1).

### Association of SMC exposure and educational attainment.

Generalized additive mixed model estimates showed a nonlinear association between SMC exposure and educational indicators (Figures 1 and 2). The marginal effect plots illustrated that 1) student enrollment increased with greater SMC exposure, particularly after 3 or more years of implementation; 2) dropout rate declined significantly after 2 or more years of SMC, reaching the lowest point around 4 years of implementation; 3) repetition rate followed a curvilinear trend with a minimum around 3 years of SMC, suggesting reduced repetition rates with moderate SMC exposure; and 4) CFEE exam pass rate showed a marked increase after 3 years of SMC, with a sharp improvement following 4 and 5 years of exposure (Figure 2). When comparing districts that had or had not been targeted for SMC, those implementing SMC consistently outperformed non-SMC districts across all education indicators analyzed, suggesting that sustained implementation of SMC improves educational outcomes in children, particularly in terms of early school participation and progression.

**Figure 1.**
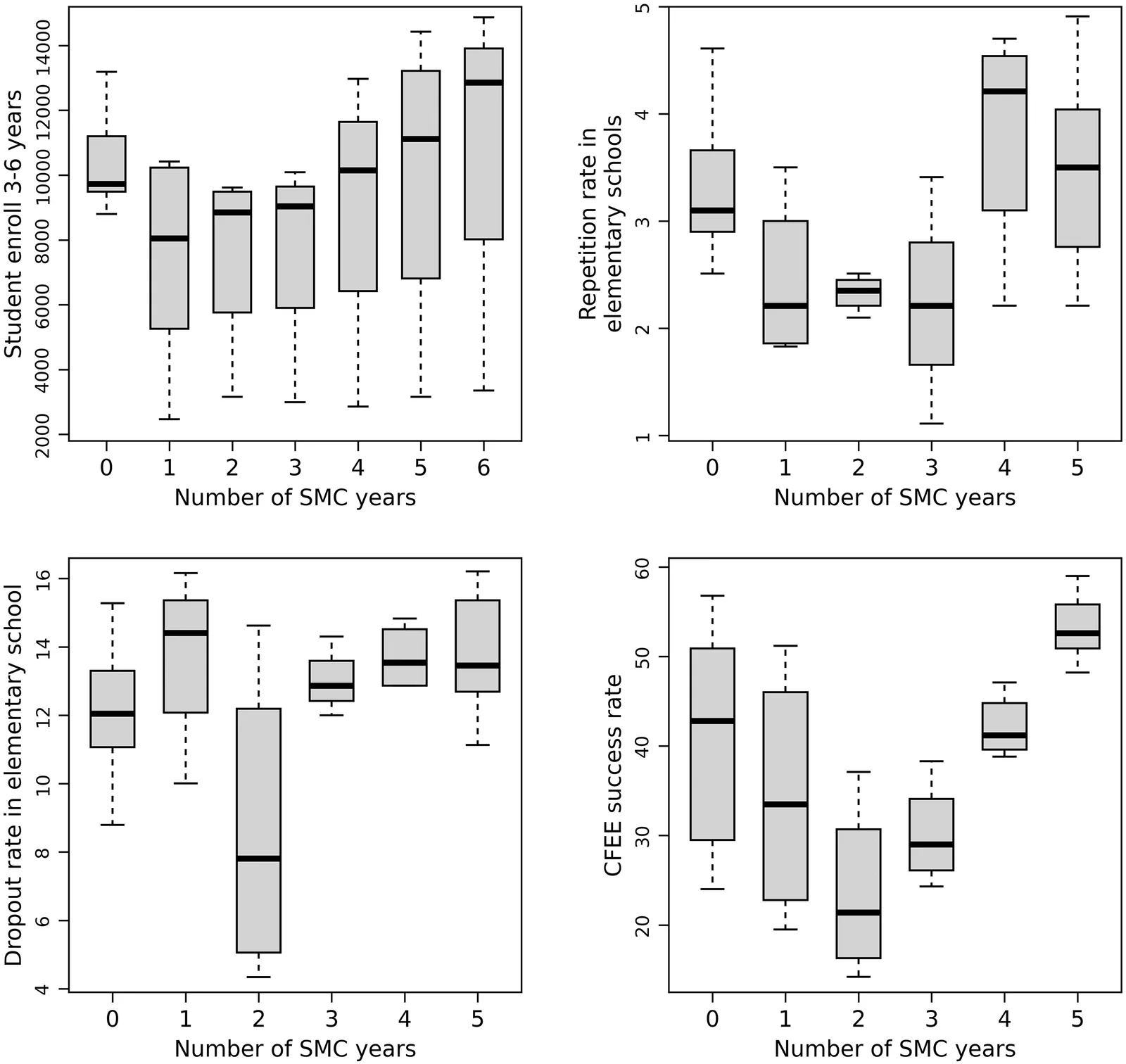
Descriptive statistics of education outcome indicators by seasonal malaria chemoprevention (SMC) exposure and non-SMC districts, Senegal, 2013–2019.

**Figure 2.**
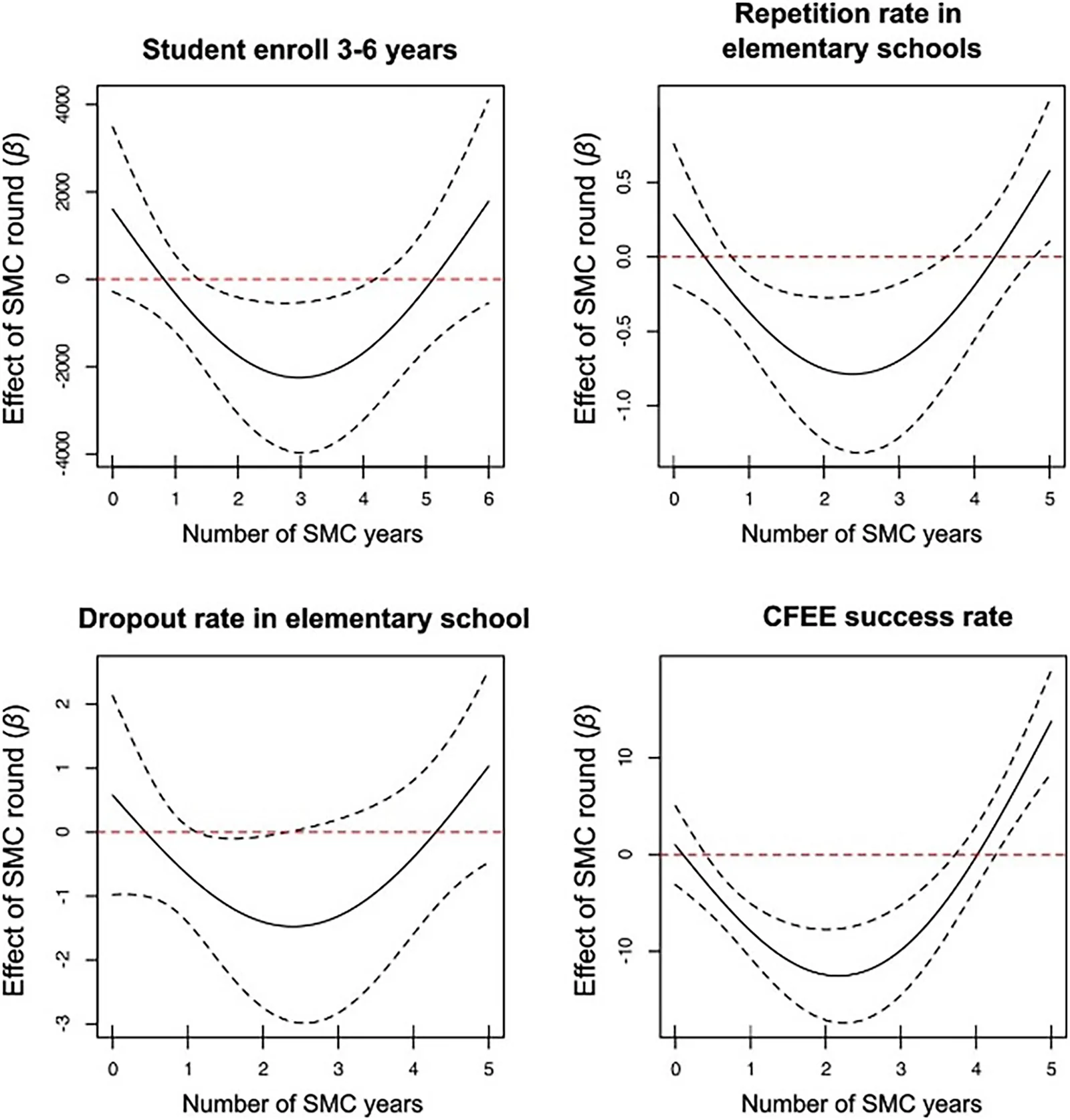
Association between number of seasonal malaria chemoprevention cycles and education outcome indicators in Senegal, 2013–2019.

## DISCUSSION

Seasonal malaria chemoprevention has been implemented in Senegal since 2013, with programmatic coverage consistently above 90%. Combined with the scale-up of other malaria interventions (i.e., community-level malaria case management, insecticide-treated nets [ITNs], enhanced surveillance), SMC has contributed to the 73% and 13% reductions in malaria case incidence and deaths observed in Senegal from 2000 to 2019, respectively.^12^ Initial analyses during the piloting of SMC in Senegal showed that it reduced the incidence of confirmed malaria cases by 60% (95% CI: 54%–64%).^13^ Similarly, a case–control study conducted in Kédougou region in 2016 showed that SMC provided 89% (95% CI: 72%–96%) protective efficacy against malaria infection in children under 10 years of age.^14^ Our results suggest that prolonged protection from malaria provided by multiple years of programmatic SMC implementation is associated with improved education. This result is consistent with longer-term retrospective studies of chemoprevention trials^15^ and programmatic use of bed nets^16^ and is, to our knowledge, the first analysis of the impact of programmatic SMC implementation on education outcomes. We also show that routine malaria intervention and education data can be used to potentially measure the impact of malaria interventions such as SMC on education outcomes. Districts that received multiple years of SMC had improved education outcomes, specifically for preschool enrollment, primary school dropout and repetition rates, and rates of passing the CFEE exam. Our study adds to the growing literature showing that malaria interventions targeted to children not only result in decreases in malaria incidence of cases and deaths but may also have a broader impact on nonhealth outcomes.^17^^–^^19^ Malaria may negatively impact education through repeated episodes of clinical disease and prolonged periods of chronic infection, both of which may result in anemia and inflammation, impairing learning by either undermining cognitive development or function (e.g., decreased sustained attention), or school absences, which—if repeated and cumulative—negatively affect education outcomes.

A couple of potential caveats in our analyses should be highlighted. First, our analyses did not adjust for heterogeneity in malaria transmission, malaria intervention coverage (e.g., ITNs), or education intervention coverage. Rather, the heterogeneity of these variables was accounted for by using a random effect in the GAMM framework, which assumes heterogeneity in these variables at the district level. Second, use of routine programmatic data can be problematic because of internal validity, completeness, and potential bias in estimates of association or effect, and caution must be exercised in interpreting analytical findings.^20^ However, since the period 2008–2010, Senegal has prioritized robust, well-governed data systems, including passing various laws that defined data systems architecture, governance, and operability. Both health and education data platforms have been digitized for the past 15 years, with data being continuously used for analysis and reporting by government and development partners. In addition, Senegal’s health and education ministries have conducted regular nationally representative surveys to complement the data being reported out of routine systems.

Future studies determining the effectiveness of SMC and other malaria control interventions should go beyond estimating the impact on children’s health and include nonhealth indicators, specifically childhood development and education outcomes, including grade progression, learning, and attainment. Measuring these broader impacts is critical to fully capturing the potential societal gains from health interventions such as SMC.
